# Learning and implementation of TransREctus sheath PrePeritoneal procedure for inguinal hernia repair

**DOI:** 10.1007/s10029-024-03031-x

**Published:** 2024-05-17

**Authors:** T. L. R. Zwols, A. R. M. van der Bilt, N. J. G. M. Veeger, M. J. W. Möllers, D. A. Hess, H. T. Brandsma, E. Jutte, P. H. J. M. Veldman, H. H. Eker, G. G. Koning, J. P. E. N. Pierie

**Affiliations:** 1 Department of Surgery, Medical Centre Leeuwarden, Leeuwarden, The Netherlands; 2 Ommelander Hospital Groningen, Scheemda, The Netherlands; 3 MCL Academy, Medical Center Leeuwarden, Leeuwarden, The Netherlands; 4 Department of Surgery, Nij Smellinghe Hospital, Drachten, The Netherlands; 5 Department of Surgery, Antonius Hospital, Sneek, The Netherlands; 6 Department of Surgery, Tjongerschans Hospital, Heerenveen, The Netherlands; 7 Department of Surgery, University Hospital, Ghent, Belgium; 8 Department of Surgery, Euregio Hospital, Nordhorn, Germany; 9 Department of Epidemiology, University of Groningen, University Medical Center Groningen, Groningen, The Netherlands; 10 Postgraduate School of Medicine, University Medical Centre Groningen, Groningen, The Netherlands

**Keywords:** TREPP, Preperitoneal, Inguinal, Hernia, Learning, Implementation

## Abstract

**Purpose:**

The TransREctus sheath PrePeritoneal procedure (TREPP) was introduced as an alternative open and preperitoneal technique for inguinal hernia mesh repair, demonstrating safety and efficacy in retro- and prospective studies. However, little is known about the technique’s inherent learning curve. In this study, we aimed to determine TREPP learning curve effects after its implementation in high-volume surgical practice.

**Methods:**

All primary, unilateral TREPP procedures performed in the first three years after implementation (between January 2016 and December 2018) were included out of a large preconstructed regional inguinal hernia database. Data were analyzed on outcome (i.e., surgical complications, hernia recurrences, postoperative pain). Learning curve effects were analyzed by assessing outcome in relation to surgeon experience.

**Results:**

In total, 422 primary, unilateral TREPP procedures were performed in 419 patients. In three patients a unilateral TREPP procedure was performed on both sides separated in time. A total of 99 surgical complications were registered in 83 procedures (19.6% of all procedures), most commonly inguinal postoperative pain (8%) and bleeding complications (7%). Hernia recurrences were observed in 17 patients (4%). No statistically significant differences on outcome were found between different surgeon experience (< 40 procedures, 40–80 procedures, > 80 procedures).

**Conclusion:**

Implementation of TREPP seems not to be associated with a notable increase of adverse events. We were not able to detect a clear learning curve limit, potentially suggesting a relatively short learning curve among already experienced hernia surgeons compared to other guideline techniques.

**Supplementary Information:**

The online version contains supplementary material available at 10.1007/s10029-024-03031-x.

## Introduction

The TransREctus sheath PrePeritoneal procedure (TREPP) has emerged as an alternative open and preperitoneal approach to inguinal hernia repair. During this procedure, the preperitoneal space is accessed by opening the anterior layer of the rectus sheath. After reduction of the medial, lateral and/or femoral hernia a mesh with a self-expanding ring is inserted covering all hernia sites [1–3]. Recent studies show comparable or more favorable outcomes than Lichtenstein repair or minimal invasive surgery (laparo-endoscopic or robotic) [4, 5]. It offers the possibility of preperitoneal mesh placement through an open but minimal invasive approach, without the need for endoscopic surgical facilities or general anesthesia risks. Other potential advantages attributed to TREPP may be lower treatment costs and its applicability in emergency surgery for strangulated inguinal hernia [6].

However, the implementation of TREPP in inguinal hernia treatment is hampered by limited experience and, thus, subject to debate. Although the body of evidence is expanding, questions still remain concerning its applicability in current surgical practice [7]. We have previously investigated that TREPP shows comparable efficacy (in terms of recurrences) and morbidity (in terms of postoperative pain and surgical site-related complications) compared to either conventional open (Lichtenstein) or endoscopic (totally extraperitoneal, TEP) inguinal hernia surgery in a large cohort [8]. Nonetheless, currently available literature does not allow any recommendation about the preferred approach to preperitoneal mesh placement, in accordance with the international guidelines for groin hernia management [9].

A major caveat both in interpreting current TREPP data as well as in implementing this novel technique in day-to-day surgical practice is our lacking understanding (knowledge) of its learning curve. It is likely that the ease of adding this technique to the surgeon’s operative armamentarium will determine its use alongside already established techniques.

The aim of this retrospective study is to gain insights in the TREPP learning curve by analyzing the occurrence of adverse events (surgical complications and hernia recurrences) and operating time—by way of proxy—during the implementation of this novel procedure in a high-volume, regional surgical collaborative.

## Materials and methods

### Study design

We previously performed a propensity-score-matched analysis of consecutive patients undergoing inguinal hernia repair in a multicenter regional surgical collaborative in the Netherlands [8]. For the current study, we included patients who underwent TREPP procedures between January 1st 2016 and December 31st 2018. In our selection, we included all primary and unilateral inguinal hernias for which TREPP was performed. Cases with bilateral inguinal hernias or recurrences were excluded. In this period, the TREPP was implemented in our surgical practice among experienced hernia surgeons. Follow-up registry was updated until November 2019, comprising data on surgical complications and hernia recurrence. In addition, procedural parameters were obtained from the operation logs, including duration of surgery and composition of the surgical team. All data were acquired and anonymized adhering to Dutch and institutional regulations. The Medical Ethics Committee of our institution (RTPO Leeuwarden) confirmed the conduct of this retrospective study without the need for ethical review, and the institutional board approved the execution of the study without the need for consent in accordance with Dutch regulations.

### Surgical procedure

All patients underwent TREPP inguinal hernia repair following nine key-steps, as described previously [1]. For all procedures, a polypropylene mesh with a memory ring was used (PolySoft Hernia patch, Bard Medical, the Netherlands, and the Rebound mesh, Duomed, the Netherlands). Procedures were performed by 14 different surgeons with extensive experience in conventional open and endoscopic hernia repair techniques. Four surgeons also had previous TREPP experience (see Supplementary Table A). Surgeons without previous TREPP experience were introduced to the technique by proctor-TREPP surgeons. The procedure was implemented subsequently in our surgical group, with other surgeons being supervised by one of these proctors during the first procedures. When the proctoring surgeon deemed the surgeon sufficient in the described 9-step technique (as described by Akkersdijk et al. [1]), this surgeon would continue with performing the procedures without supervision.

### Outcome measures

Postoperative follow-up at the outpatient clinic was performed routinely at 2–6 weeks after surgery. Additional visits were scheduled in case of adverse events, or after subsequent referral. Charts were reviewed for information on several procedure-related adverse events—including postoperative pain, surgical site infection, hematoma/bleeding, seroma formation—and hernia recurrence. Postoperative pain was defined as any registrations in the patient file of additional outpatient clinic evaluation after regular follow-up due to complaints of inguinal pain or by receiving additional diagnostic studies, analgesic treatment or referral to a pain specialist [8], Furthermore, operating time (i.e., time between incision and wound closure) and composition of surgical team (as procedures during the early implementation period were more often performed by two surgeons) were registered from the operation logs.

Adverse events (defined as both surgical complications—i.e., bleeding, infectious sequelae, postoperative pain and seroma formation—as well as hernia recurrence) were used as proxies to determine learning curve effects in keeping with similar studies [10], by analyzing their temporal incidence based on ranking consecutive procedures per surgeon.

### Statistical analysis

Patient and procedural characteristics are displayed using descriptive statistics. Chi-square and two-tailed Fisher’s exact tests were used to analyze the relationships between categorical variables, to which end data were dichotomized as described explicitly in text and tables. To analyze the influence of increasing experience on our outcome variables, we plotted the occurrence of adverse events in relation to increasing surgeon experience for TREPP procedures. Surgeons were categorized based on experience, for which comparisons were performed using the Kruskal–Wallis test and cross-tabulation analyses as described above. All *p* values<0.05 (two sided) were considered statistically significant. All statistical analyses were performed using SPSS 24 (IBM, the Netherlands) and SAS 9.4 software (SAS Institute Inc., Cary, North Carolina, USA).

## Results

### Procedural characteristics

**Fig. 1:**
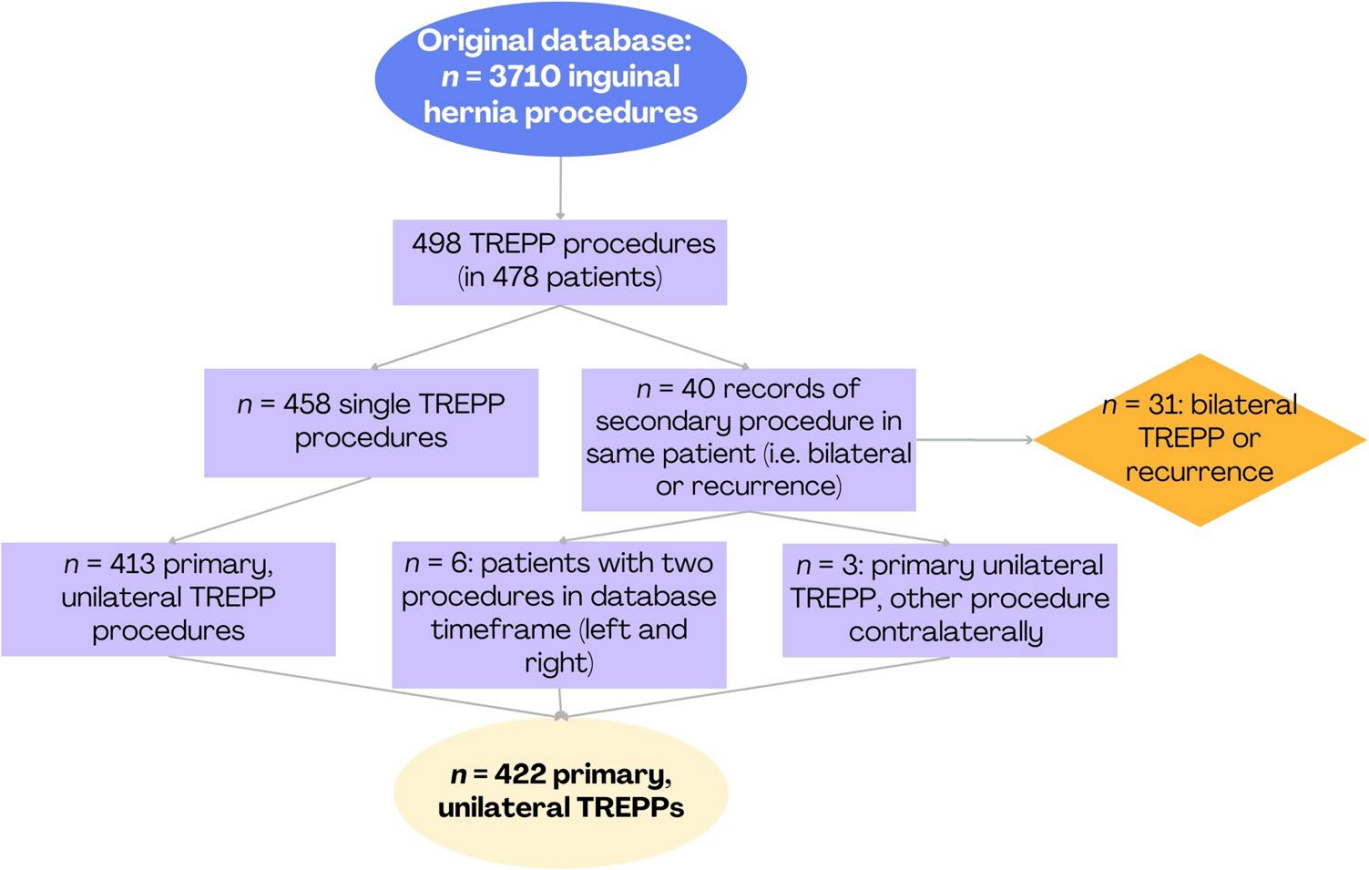
Flowchart showing selection of procedures of large retrospective cohort study

We overviewed data from 422 TREPP procedures in 419 patients, as shown in Fig. 1. Out of all cases, only primary, unilateral TREPPs were included. Table 1 shows patient and procedural characteristics.

**Table 1:** Procedural and patient characteristics

Characteristic	*n*	(%)	Years/min^a^
Total procedures	422		
Male	405	(96)	
Female	17	(4)	
Age			
Median			58
Range			18–88
ASA classification			
1	171	(41)	
2	261	(51)	
3	35	(8)	
4	0	(0)	
Urgency			
Elective	419	(99)	
Emergency	3	(1)	
Operating time^b^			
Median			21
Range			9–70

^a^Age displayed in years, duration of operative procedure displayed in minutes

^b^Based on data present in 271 out of 422 cases

The characteristics were representative for a general inguinal hernia cohort, with a male predominance, in general favorable ASA scores and median age at surgery of 58 years. TREPPs were mostly performed electively for primary, unilateral inguinal hernias. Median operating time was 21 min (range 9–70).

### Adverse events

Table 2 summarizes the TREPP-related surgical complications and hernia recurrences—taken together as adverse events—observed in our cohort. A total of 94 surgical complications were registered, most commonly bleeding complications (6.6%) and postoperative inguinal pain (7.8%). The hernia recurrence rate was 4.0%.

**Table 2:** Procedure-related adverse events

Procedure-related adverse events	*n*	(%)
Bleeding^a^	28	(6.6)
Seroma	9	(2.1)
Infection^b^	7	(1.7)
Postoperative pain	33	(7.8)
Recurrence	17	(4.0)

^a^Comprised of presence of hematoma (*n* = 26) and/or postoperative bleeding (*n *= 5)

^b^Comprised of site infection (*n* = 7) and/or abscess (*n *= 2)

### Adverse events in relation to surgeon experience

**Fig. 2:**
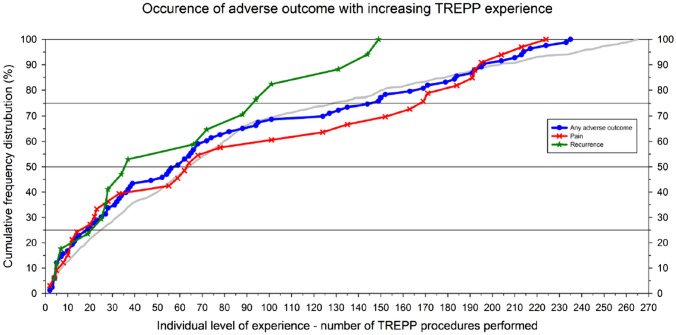
Adverse events with increasing TREPP experience. The occurrence of events (vertical axis, set to 100%) is shown in relation to increasing surgeon experience defined by procedure rank number (horizontal axis)

To further investigate the presence and magnitude of a learning curve effect on the occurrence of adverse events, data were analyzed sequentially by designating a rank number to each procedure performed per surgeon, thus reflecting the level of experience per surgeon. We considered previous experience (Supplementary Table A) of four surgeons with previous TREPP experience. Figure 2 shows the occurrence of adverse events in function of increasing TREPP experience. We corrected for previous TREPP experience when relevant, with lowest case numbers corresponding with surgeons new to this technique. Procedures performed by more experienced surgeons have higher case numbers. Adverse events are represented by the blue line. Recurrences are displayed by the green line.

Considering the lack of previous data on TREPP learning curve numbers, experience was divided based on tertiles, making experience groups of 40: little experience (<40 procedures), medium experience (40–80 procedures) and much experience (>80 procedures). This is consistent with learning curve studies for other inguinal hernia techniques that used cut off point at 30–35 procedures. Results in these groups are shown in Table 3. We did not observe a significantly higher overall incidence of adverse events among TREPPs performed by hernia surgeons with little TREPP experience, compared to their counterparts with much TREPP experience before implementation of the technique in our surgical practice. Recurrence rates were 3.5% (most experience) *vs* 2.0% (medium experience) *vs* 6.0% (no experience), *p *= 0.26. Postoperative pain was present in 8.2% of cases in the experienced group, compared to 5.9% (medium experience) and 8.7% (no experience), *p *= 0.71. We observed a statistically significant difference in operating time, being the shortest in the most experienced group; 21.6 (± 9.5) minutes (*p *= 0.001).

**Table 3:** Outcome in relation to surgeon TREPP experience

	Experience		
	Group 1: *n* > 80^a^	Group 2: *n* = 40–80	Group 3: *n *= 0–40
Adverse events	18.1%	15.8%	24.0%
RR (95% CI)^b^		0.9 (0.5–1.5)	1.3 (0.9–2.0)
Recurrence	3.5%	2.0%	6.0%
RR (95% CI)		0.6 (0.1–2.7)	1.7 (0.6–4.7)
Postoperative pain	8.2%	5.9%	8.7%
RR (95% CI)		0.7 (0.3–1.8)	1.1 (0.5–2.2)

^a^*n* = number of procedures

^b^Relative risks are compared to the most experienced group (group 1)

## Discussion

TREPP seems to offer a promising alternative to more widely performed inguinal hernia mesh repairs, mainly endoscopic preperitoneal (TEP, TAPP) or open anterior (Lichtenstein) techniques. It enables preperitoneal mesh placement without the need for risk associated with general anesthesia or advanced technological resources. Previous large cohort studies show similar incidences of postoperative pain and recurrences when comparing TREPP to TEP or Lichtenstein [8, 11], while showing a significantly shorter operating time for TREPP [9, 11–13]. However, its place in the contemporary surgical practice has yet to be determined, and will likely depend on the ease with which the procedure can be learned and implemented [14, 15]. As stated by the HerniaSurge Group, there is a need for learning curve studies to determine the value of open preperitoneal mesh techniques [9].

This is currently the first study addressing a possible TREPP learning curve in common daily practice among experienced hernia surgeons. In our retrospective cohort, we analyzed the occurrence of adverse events (both surgical complications and inguinal hernia recurrences) in relation to surgeon experience with TREPP in 422 primary unilateral TREPP procedures (in 419 patients) with increasing level of TREPP procedure experience. These parameters are frequently used as proxies for learning curve effects, and have been widely used and validated in previous studies assessing the learning curves of various other hernia repair techniques [9, 16–18]. We conclude that the implementation of TREPP is not associated with a notable increase of adverse events, with outcomes comparable to known literature and no clear dependence on previous TREPP experience.

To our knowledge, this cohort is one of the largest available with physician-reported outcomes for TREPP surgery [6, 8]. One large retrospective study performed by Lange et al. described patient-reported (questionnaire) outcomes in 932 patients after TREPP, reporting 8% postoperative pain and 1–5% recurrence [2]. Both pain and recurrence have been primarily used as primary endpoints to describe the efficacy of TREPP. A recent randomized-controlled trial (ENTREPPMENT trial) comparing TREPP with transinguinal preperitoneal (TIPP) mesh repair reported inguinal pain in 7.2% and recurrence in 8.9% out of 400 patients one year after TREPP [14]. We assess a comparable 7.8% incidence of postoperative inguinal pain which is comparable to previous literature, as well as a 4% recurrence rate.

Although others have hypothesized that the TREPP learning curve could be favorable, total data were too small to support or reject this claim [3, 19]. Bökkerink et al. have recently shown in data from the ENTREPPMENT trial that the relatively high incidence of recurrences observed after TREPP was largely attributed to procedures performed by inexperienced TREPP surgeons [14]. After disregarding the first 10 procedures performed per surgeon, the recurrence rate dropped from 8.9% to 6.7%. Among the most experienced surgeons participating in that trial, they describe an even lower rate of 2.1% recurrences. However, no quantification is available for the length of the TREPP learning curve or the approximate number of proctored and/or supervised procedures needed by the individual surgeon to flatten the learning curve.

We hypothesized a learning curve of less than 40 procedures, but did not reach statistical significance in our analysis. This is likely caused by the inherently limited complication rate of inguinal hernia surgery, thus requiring a very large sample size for adequate statistical power, and the retrospective nature of our study. Nevertheless, the TREPP learning curve seems to compare favorably to its endoscopic counterpart. For the TEP procedure extensive clinical experience exists and several studies have been conducted analyzing its learning curve. Estimates between 50 and 100 procedures have been postulated for the TEP learning curve [20–23], but also upwards of 250–400 procedures results may further improve significantly before reaching a steady state [24].

It could be hypothesized that the apparently steep TREPP learning curve benefits from the generally shorter learning curves of open compared to endoscopic surgery. Another hypothesis could be the anatomical insight already available to surgeons with endoscopic experience. Therefore, it would be of great interest to analyze TREPP outcomes and learning curve effects among surgeons less or unfamiliar with endoscopic repair (i.e., residents or surgeons in developing countries). Although further research is suggested, our current data point out that the TREPP technique could be safely and expeditiously adapted into clinical practice.

### Supplementary Information

Below is the link to the electronic supplementary material.

Supplementary file1 (DOCX 22 KB)
