# Supplementary material for: Learning and implementation of TransREctus sheath PrePeritoneal procedure for inguinal hernia repair
Source: Hernia. 2024 May 17;28(4):1309–15. doi: 10.1007/s10029-024-03031-x (PMC11297057; doi:10.1007/s10029-024-03031-x)
Supplement: Supplementary file 1 — Supplementary file1 (DOCX 22 KB) [file 10029_2024_3031_MOESM1_ESM.docx]

| Surgeon | Experience pre-study | Procedures in study | Complication | Events (by rank number) | | | | |  |  |  |  |  |  |  |  |  | Total |
| --- | --- | --- | --- | --- | --- | --- | --- | --- | --- | --- | --- | --- | --- | --- | --- | --- | --- | --- |
| 1 | 100 | 122 | Pain | 101 | 124 | 135 | 152 | 163 | 169 | 171 | 184 | 191 | 192 | 195 | 204 | 213 | 224 | 14 |
| 1 |  |  | Bleeding | 127 | 171 | 196 | 214 | *217* | 224 |  |  |  |  |  |  |  |  | 6 |
| 1 |  |  | Infection | 127 | 183 | 195 | 210 | 235 |  |  |  |  |  |  |  |  |  | 5 |
| 1 |  |  | Seroma | 131 | 179 | 192 | 217 | 233 |  |  |  |  |  |  |  |  |  | 5 |
| 1 |  |  | Recurrence | 101 | 131 |  |  |  |  |  |  |  |  |  |  |  |  | 2 |
| 2 | 0 | 91 | Pain | 10 | 12 | 20 | 23 | 64 | 78 |  |  |  |  |  |  |  |  | 6 |
| 2 |  |  | Bleeding | 5 | 13 | 19 | 38 | 39 | 47 | 54 | 56 | 62 | 65 | 74 | 82 | 95 |  | 13 |
| 2 |  |  | Seroma | 13 | 52 | 68 |  |  |  |  |  |  |  |  |  |  |  | 3 |
| 2 |  |  | Recurrence | 25 | 37 | 66 | 72 | 88 | 94 |  |  |  |  |  |  |  |  | 6 |
| 3 | 30 | 57 | Pain | 62 | 68 |  |  |  |  |  |  |  |  |  |  |  |  | 2 |
| 4 | 50 | 37 | Pain | 55 | 59 |  |  |  |  |  |  |  |  |  |  |  |  | 2 |
| 4 |  |  | Seroma | 59 |  |  |  |  |  |  |  |  |  |  |  |  |  | 1 |
| 5 | 0 | 35 | Pain | 2 |  |  |  |  |  |  |  |  |  |  |  |  |  | 1 |
| 5 |  |  | Bleeding | 36 |  |  |  |  |  |  |  |  |  |  |  |  |  | 1 |
| 5 |  |  | Infection | 5 |  |  |  |  |  |  |  |  |  |  |  |  |  | 1 |
| 5 |  |  | Recurrence | 7 | 28 |  |  |  |  |  |  |  |  |  |  |  |  | 2 |
| 6 | 0 | 33 | Pain | 8 | 14 | 28 | 33 |  |  |  |  |  |  |  |  |  |  | 4 |
| 6 |  |  | Bleeding | 31 | 32 |  |  |  |  |  |  |  |  |  |  |  |  | 2 |
| 6 |  |  | Recurrence | 19 | 27 | 34 |  |  |  |  |  |  |  |  |  |  |  | 3 |
| 7 | 0 | 20 | Pain | 4 | 5 | 12 | 22 |  |  |  |  |  |  |  |  |  |  | 4 |
| 7 |  |  | Bleeding | 7 | 15 |  |  |  |  |  |  |  |  |  |  |  |  | 2 |
| 8 | 0 | 8 | Bleeding | 3 |  |  |  |  |  |  |  |  |  |  |  |  |  | 1 |
| 8 |  |  | Infection | 3 |  |  |  |  |  |  |  |  |  |  |  |  |  | 1 |
| 8 |  |  | Recurrence | 4 | 5 |  |  |  |  |  |  |  |  |  |  |  |  | 2 |
| 9 | 140 | 8 | Bleeding | 150 |  |  |  |  |  |  |  |  |  |  |  |  |  | 1 |
| 9 |  |  | Recurrence | 144 | 149 |  |  |  |  |  |  |  |  |  |  |  |  | 2 |
| 11 | 0 | 3 | Bleeding | 4 | 5 |  |  |  |  |  |  |  |  |  |  |  |  | 2 |

**Supplementary Table A - occurrences of complications per surgeon experience rank number. Occurrences in same patient are underlined.**
